# Strengthening financial management systems at primary health care: Performance assessment of the Facility Financial Accounting and Reporting System (FFARS) in Tanzania

**DOI:** 10.3389/frhs.2022.787940

**Published:** 2023-01-09

**Authors:** George M. Ruhago, Frida N. Ngalesoni, Ntuli A. Kapologwe, James T. Kengia, James Ngocho, Stephen M. Kabusi, Albino Kalolo, Erick J. Kitali, Elisa Rwamiago, Gemini Mtei

**Affiliations:** ^1^ Department of Development Studies, School of Public Health and Social Sciences, Muhimbili University of Health and Allied Sciences, Dar Es Salaam, Tanzania; ^2^ Amref Health Africa, Dar Es Salaam, Tanzania; ^3^ President's Office Regional Administration and Local Government (PORALG), Dodoma, Tanzania; ^4^ Institute of Public Health, Kilimanjaro Christian Medical University College, Moshi, Tanzania; ^5^ Department of Public Health, School of Nursing and Public Health, University of Dodoma, Dodoma, Tanzania; ^6^ Department of Public Health, St Francis University College of Health and Allied Sciences, Ifakara, Tanzania; ^7^ Abt Associates, Tanzania Public Sector Systems Strengthening Plus (PS3+) Project, Dar Es Salaam, Tanzania

**Keywords:** Facility Financial Accounting and Reporting System, facility financial management, health facility expenditure, health facility revenue, primary health care, universal health coverage, Tanzania

## Abstract

**Background:**

Universal coverage remains a challenging pursuit around the world, even among the highest-income countries. Strengthening financial management capacity is essential towards attaining the three universal health coverage (UHC) goals, namely, expanded coverage, quality service, and financial protection. In this regard, Tanzania introduced the Facility Financial Accounting and Reporting System (FFARS) in line with the introduction of the Direct Health Facility Financing (DHFF) initiative in primary health care (PHC) in 2017–2018. We aim to assess the functionality of the FFARS in management, accounting, and reporting funds received and disbursed in the stride forward strengthening public financial management in PHC facilities towards UHC.

**Methods:**

The study applied implementation research using a concurrent convergent mixed-methods design to assess sources of revenue, expenditure priorities, and changes of revenues and to explore the usability and benefits of FFARS in improving facility finance and reporting systems in more than 5,000 PHC facilities in Tanzania. Quantitative methods assessed the changes in revenues and expenditure between the financial years (FYs) 2017–2018 and 2018–2019, while the qualitative part explored the usability and the benefits FFARS offers in improving facility finances and reporting systems. Data analysis involved a thematic and descriptive analysis for qualitative and quantitative data, respectively.

**Results:**

Of the 5,473 PHC facilities, 88% were in rural areas; however, the annual average revenue was higher in urban facilities in FYs 2017–2018 and 2018–2019. Overall, district hospitals showed an increase whereas health centers reported a decline of more than 40% in revenue. The user fee was the predominant source of revenue, particularly in urban facilities, while revenue from health insurance was not among the top three highest sources of revenue. Expenditure priorities leaned more towards drugs and supplies (25%) followed by allowances and training (21%); these did not differ by facility geographies. In health centers, expenditure on facility infrastructure was predominant. Key Informant Interviews revealed an overall satisfaction and positive experiences related to the system.

**Conclusion:**

The implementation of FFARS in Tanzania demonstrated its high potential in improving facility financial management, including its ability to track revenue and expenditure at PHC facilities. Staffing shortages, ICT infrastructure, and limited opportunities for capacity building could be the limiting factors to reaching the potential of the implementation of FFARS and the attainment of its full impact on Tanzania's pursuit for UHC.

## Background

Progress towards universal health coverage (UHC) requires advancement on the three UHC goals, which includes improving coverage in the use of health services, quality service, and financial protection. Health system financing arrangements are crucial in achieving these goals through making health systems more transparent, accountable, efficient, and people centered (1, 2). The World Health Organization (WHO) points out that the impediment to a more rapid movement towards UHC is the inefficient use of resources (3, 4). The efficient use of resources reduces waste, improves the ability of health systems to provide quality services, and improves population health. The financial resources represent one of the vital healthcare system inputs.

It has been noted that decentralization reforms do not necessarily result in improved funding flows, finance management autonomy, or accountability mechanisms, and for public hospitals, it is irrespective of the mode and form of decentralization reform adopted (5). Tanzania, therefore, has in the last decade made progress in strengthening the health system financing with progress towards UHC. The major efforts in strengthening the health financing system include development of the health financing strategy, reforming the community health fund (CHF), and the introduction of direct health facility financing (DHFF). DHFF was introduced in all district councils in the FY 2017–2018 (3). DHFF is an extension to local government reforms of decentralization by devolution (D by D), a fiscal decentralization of financial resources directly to health facilities to improve health system performance by linking payment to priority service, enhancement of autonomy, transparency, and accountability at the facility level.

To enable the operationalization of DHFF, a financial and accounting system known as the Facility Financial and Reporting System (FFARS) was developed. FFARS is an application that allows the recording of budget disbursement, expenditure, and the generating of reports at the facility, council, regional, and national levels. The system was launched after a comprehensive consultative interaction from design to implementation including various stakeholders, such as the President's Office Regional Administration and Local Government (PORALG), Ministry of Finance and Planning (MoFP), Ministry of Health (MOH), Ministry of Education, Science and Technology (MOEST), Local Government Authorities (LGAs), regional and local government authorities, and lower facilities in collaboration with the United States Agency for International Development (USAID)-funded project, the Public Sector Systems Strengthening (PS3).

The FFARS conformed to the requirement of accounting standards, such as the IPSAS 35, standards that demand consolidated financial statements that include accounts of all levels in the LGAs. In line with this, the classification of income and expenditure items in the FFARS is the same at health facilities and LGA levels. Hence budget codes used at the council level also apply at the facility level, i.e., cost center codes, fund types, and same the Government Finance Statistics Manual (GFS) codes for inputs that are based on the chart of accounts approved by the MoFP. The FFARS provides an electronic version of facility-level financial and accounting information, serves data collected to a database, and it operates on mobile devices using the android application system and through a web application.

The system is currently active in over 5,473 health facilities, enabling all levels of the government to transparently and efficiently manage, account, and report funds received and disbursed. FFARS, which is interoperable with the planning and budgeting tool (PlanRep), described elsewhere (6), makes up the public financial management systems, both of which are extended to the health facility level through D by D reforms. To date, there is a paucity of information on how electronic financial and accounting tools are taken into real operation in primary healthcare (PHC) settings. The aim of the present study was to assess the functionality of the FFARS in relation to management, accounting, and reporting funds received and disbursed in the stride forward to strengthening public financial management in PHC facilities within the context of the realization of the UHC goal.

## Methods

### Study area and design

Tanzania is a lower middle-income country located in East Africa with a population of 61,627,284. Tanzania has had a steady economic growth in the range of 5%–7% before COVID-19 but fell to 4.3% in 2022 (9). In the attempt to reach UHC, Tanzania has made tremendous progress in increasing coverage of health services, which has translated into an improvement in some health indicators, such as under-five mortality that has been reduced by 44.2% between 1990 and 2017 (7), and shown efforts in the introduction and sustaining of prepayment schemes, including the forthcoming universal health insurance as a means of financial protection. To sustain these efforts and avert health system challenges, there is a high need to invest in evidence-based planning and financial accounting using a bottom-up approach.

The present study employed a concurrent convergent mixed-methods design to assess sources of revenue, expenditure priorities, and changes of revenues and to explore the usability and benefits of FFARS in improving facility finance and reporting systems. The convergent concurrent mixed-methods design entails collecting both qualitative and quantitative data at the same time (in a parallel manner) to answer the research questions (8). We assessed sources of revenue, expenditure priorities, and the changes of the revenues collected between the FYs 2017–2018 and 2018–2019 while also exploring the usability, functionality, and benefits FFARS offers in improving facility finances and reporting systems. Furthermore, we explored experiences in recording financial transactions, e.g., payment systems and procedures, recording of revenue and expenditure, bank reconciliation, procurement aspects, and procedures for preparing financial reports. In combining the quantitative and qualitative data, we aimed to ascertain complementarity or divergence in the quest of triangulating the data to get a holistic picture of the implementation of FARRS in PHC facilities.

### Sampling procedures and sample size

The quantitative sample size included 5,183 and 5,407 health facilities in FYs 2017–2018 and 2018–2019 implementing FFARS, respectively. There were more health facilities in FY 2018–2019 due to the construction of new PHC facilities (9). The qualitative sample included key informants selected on virtue of their positions and experiences in financial management and accounting in the public PHC facility settings.

### Data collection tools and procedures

Quantitative data were extracted from the FFARS for all public health facilities implementing the system, while qualitative data involved in-depth and key informant interviews (KIIs). A total of 27 KIIs were done virtually by phone due to the COVID-19 pandemic with officials from LGAs and regional authorities (eight interviews), supporting implementing partner, PORALG, from the Health, Education, Policy Planning, Finance, and Information and Communication Technology (ICT) departments and service delivery points (heads of health facilities and schools). The interviewees had experience in using the FFARS to build an understanding of the extent of the gains that have been achieved from its use since its inception. We used a developed semi-structured interview guide for the interviews. Our interviews focused particularly on exploring the end-users’ experiences on utilizing the FFARS in comparison with the old paper-based financial management and accounting system. Specific themes explored included efficiency, utilization, enabling the environment, and sustainability in using the system. The interview explored questions around process speed, ease of operation, user-friendliness, communication, perceived cost and time saving, and the overall benefits of the systems. The pre-testing of the tools took place before the actual data collection.

### Data management and analysis

The key variables of interest in this study included sources of revenue and types of expenditure. Quantitative data were entered into Microsoft Excel sequentially and cleaned and analyzed. Sources of revenue and types of expenditure were compared in the two FYs and as a proportion of the total. Qualitative data were analyzed through thematic analysis by Braun and Clarke (10). Data were transcribed and the initial familiarization was carried out through reading and rereading the texts. Initial codes were then developed, from which themes and subthemes were developed.

### Integrated interpretation

Using a triangulation approach and aiming to obtain a holistic picture of the results, we integrated the quantitative and qualitative findings at an interpretation level. We looked for convergence, complementarity of information, or contradiction. Integration took place after the completion of data analysis.

## Results

In both FYs 2017–2018 and 2018–2019, urban facilities recorded a higher annual average revenue compared to rural facilities, despite urban facilities making up only 12% of all facilities. Trends in revenue between the 2 years show an increase in revenue at hospital levels but a decline in health centers (by 41%) and dispensaries (by 5%). Rural–urban differences are also observed more in rural facilities, with an increase in revenue in rural hospitals and more of a decline in revenue in rural health centers and dispensaries (Table 1).

**Table 1:** Annual revenues by facility level and facility geographies in 2017/2018–2018/2019.

Source of revenue	Annual revenues per facilities by source in USD											
	Hospitals				Health centers				Dispensaries			
	Urban		Rural		Urban		Rural		Urban		Rural	
	2017/2018	2018/2019	2017/2018	2018/2019	2017/2018	2018/2019	2017/2018	2018/2019	2017/2018	2018/2019	2017/2018	2018/2019
User fees	140,570 (50%)	136,824 (40%)	37,589 (18%)	34,803 (12%)	37,558 (25%)	29,252 (28%)	4,539 (4%)	3,189 (6%)	4,786 (28%)	4,110 (25%)	576 (7%)	291 (4%)
Insurance	81,719 (29%)	91,340 (27%)	71,404 (35%)	66,301 (23%)	21,762 (14%)	16,676 (16%)	5,701 (6%)	4,462 (9%)	1,875 (11%)	1,532 (9%)	1,755 (22%)	902 (12%)
HSBF	42,973 (15%)	52,717 (16%)	57,341 (28%)	61,715 (21%)	28,520 (19%)	32,280 (31%)	22,229 (22%)	21,572 (43%)	6,299 (37%)	6,603 (40%)	3,099 (39%)	2,935 (40%)
RBF	13,721 (5%)	12,590 (4%)	22,342 (11%)	29,057 (10%)	3,720 (2%)	4,701 (4%)	4,129 (4%)	5,574 (11%)	1,431 (8%)	1,424 (9%)	1,381 (18%)	1,768 (24%)
D-Grants	3,296 (1%)	16,284 (5%)	12,091 (6%)	55,158 (19%)	42,873 (28%)	14,726 (14%)	46,621 (46%)	13,771 (27%)	1,852 (11%)	2,128 (13%)	799 (10%)	947 (13%)
Others	–	28,806 (9%)	4,902 (2%)	46,338 (16%)	18,705 (12%)	6,930 (7%)	19,119 (19%)	1,735 (3%)	869 (5%)	613 (4%)	277 (4%)	567 (8%)
Total	280,683	338,562	205,670	293,371	153,138	104,564	102,338	50,303	17,112	16,411	7,886	7,412
Number of facilities	20	21	53	54	84	91	433	446	541	579	4,052	4,216

HSBF, health sector basket funds; RBF, result-based financing.

The sources of revenue reported included user fees, insurance, health sector basket funds (HSBF), results-based financing, development grants, and others. In urban hospitals and health centers, user fees have for both years predominantly represented the highest percentage of the total facility revenue (range 26%–45%), while in other facility levels, HSBF predominated, taking up more than a quarter share to the total facility revenue.

**Figure 1:**
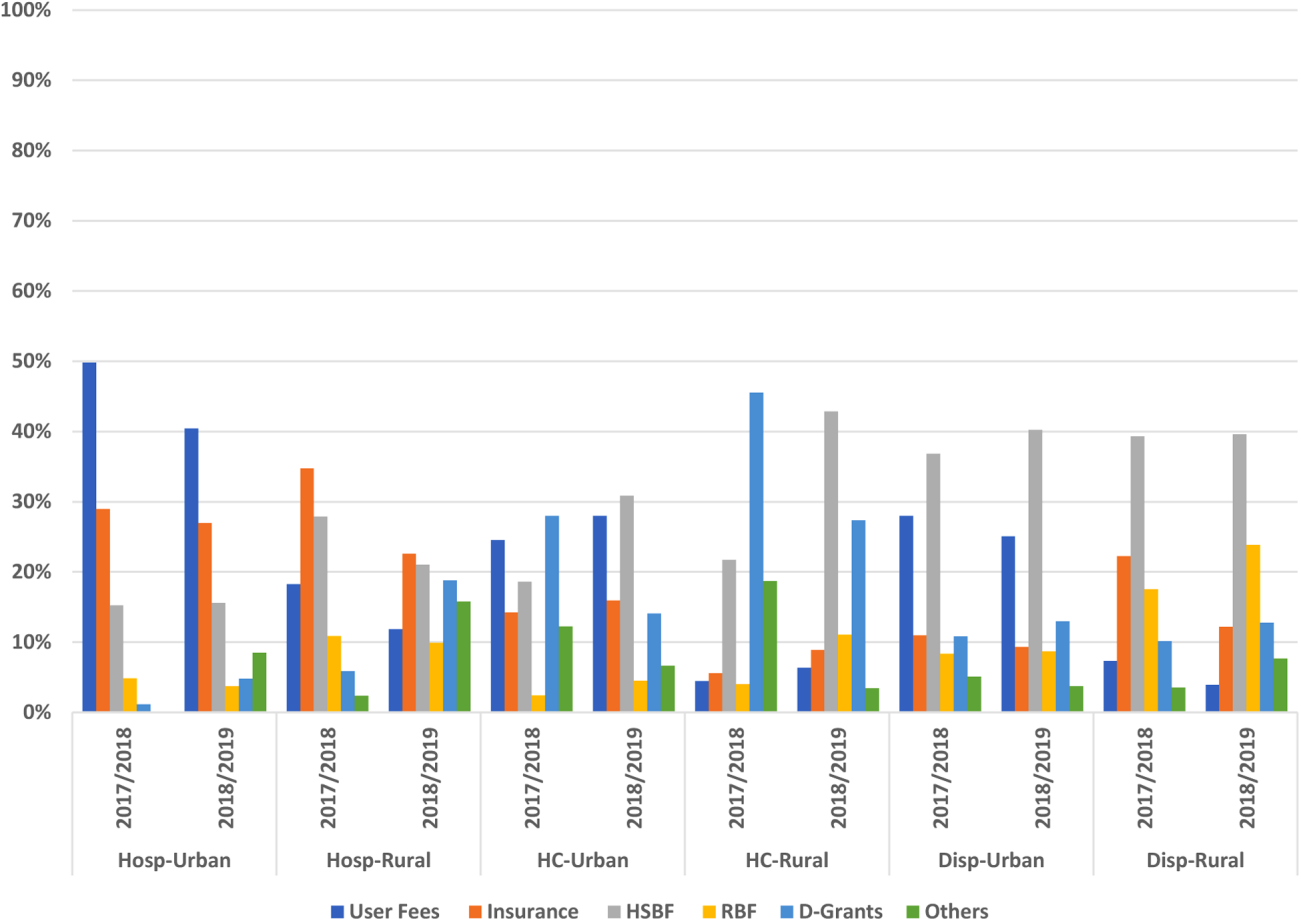
Contribution of different sources to the total revenue.

With the exception of hospitals, revenue from insurance was not among the top three highest sources of revenue with no clear district differences in facility geographical setting. Health centers reported more development grants compared to other facility levels. Overall, we observed a pattern of decrease in the contribution of user fees and insurance to the total revenue. Of note is the decrease in user fees observed among urban hospitals and the decrease in the contribution of insurance among rural dispensaries while health centers saw an increase in contribution of HSBFs to the total revenue in both urban and rural settings (Figure 1).

As with revenue, the average annual expenditure was higher in urban facilities compared to rural facilities, with an increasing trend in expenditure across all facility levels in both urban and rural settings, with the exception of rural health centers.

Even though the system still portrays low expenditure in rural facilities, the condition seemed to have been worse before the implementation of the FFARS, as narrated by this quote:

I have worked in urban and rural areas, revenue is low in rural areas compared to urban. Formerly, rural area LGAs could allocate and utilize funds that were earlier allocated for lower-level health facilities for other activities at LGA level, but now that the funds are going directly sent into the facilities accounts that cannot be done. Participant LGA

With this progress in fund flow to rural facilities, the system has brought value in reporting. A respondent from PORALG reflected this during the interview:

In the past, it was difficult to consolidate the data, we had to request reports from the districts and it could take up to 3 weeks before getting the report, now we can view this in real time from HQ.

The recording or tracking of expenditure even from the users was indicated to be important, as indicated in the quote below:

FFARS simplifies a lot because there it simplifies to see how money utilization goes, also simply it helps to see what is remaining because when you send you see directly how the expenditure goes compared to your activities and that money is used according to the budget that you planned. For example, maybe in a budget you planned to buy medicines worth 3 or 4 million, so when you deduct it from your system it shows directly that this fund has done this work directly through this activity. So, it is simple to see the fund has done what activity.

Nevertheless, full utilization of the system (and correct reflection of revenue and expenditure) requires sufficient human resources, as indicated by one LGA respondent in this quote:

The district has employed accountants, they assist dispensaries, but we have only four of these accountants, they have to oversee about 100 facilities.

We report six categories of expenditure, namely, construction and rehabilitation, drugs and medicines, medical supplies and equipment, allowances and training, vehicles and utilities, and other expenses, including office furniture and supplies (Table 2).

**Table 2:** Annual expenditure by facility level and facility geographies in 2017/2018–2018/2019.

Expenditure areas	Annual expenditure per facilities by expenditure area in USD											
	Hospitals				Health centers				Dispensaries			
	Urban		Rural		Urban		Rural		Urban		Rural	
	2017/2018	2018/2019	2017/2018	2018/2019	2017/2018	2018/2019	2017/2018	2018/2019	2017/2018	2018/2019	2017/2018	2018/2019
Infrastructure	16,845 (6%)	41,234 (12%)	14,747 (8%)	57,853 (23%)	38,600 (32%)	44,657 (36%)	41,436 (52%)	27,344 (45%)	2,891 (21%)	3,584 (26%)	1,250 (22%)	1,837 (28%)
Drugs and medicines	69,462 (26%)	87,726 (25%)	48,135 (27%)	58,097 (23%)	21,380 (18%)	22,852 (19%)	7,378 (9%)	7,697 (13%)	2,832 (21%)	2,761 (20%)	1,189 (20%)	1,199 (19%)
Medical supplies and equipment	48,002 (18%)	48,426 (14%)	24,913 (14%)	32,834 (13%)	13,864 (12%)	11,281 (9%)	6,357 (8%)	7,008 (12%)	1,489 (11%)	442 (3%)	610 (11%)	571 (9%)
Allowances and training	61,396 (23%)	81,345 (23%)	38,570 (22%)	42,663 (17%)	13,653 (11%)	18,791 (15%)	5,615 (7%)	8,163 (13%)	2,523 (18%)	3,303 (24%)	1,285 (22%)	1,663 (26%)
Vehicles and utilities	25,534 (10%)	41,832 (12%)	19,733 (11%)	27,688 (11%)	6,192 (5%)	9,309 (8%)	3,672 (5%)	5,205 (9%)	651 (5%)	1,216 (9%)	292 (5%)	375 (6%)
Others	44,276 (17%)	47,943 (14%)	32,313 (18%)	28,513 (12%)	25,310 (21%)	15,787 (13%)	14,702 (19%)	5,469 (9%)	3,259 (24%)	2,590 (19%)	1,183 (20%)	828 (13%)
Total	265,516	348,507	178,411	247,648	119,000	122,676	79,160	60,886	13,645	13,896	5,809	6,472
Number of facilities	20	21	53	54	84	91	433	446	541	579	4,052	4,216

Different health facility levels demonstrated different expenditure priorities, with the largest share of funds being spent on drugs and supplies (25%) followed by allowances and trainings (21%) for hospitals while health centers and dispensaries used 42% and 24% of their total expenses on health facility infrastructure followed by allowances and trainings, respectively.

Expenditure priorities did not differ much with facility settings except for a few instances where in the FY 2017–2018 rural health centers used 9% of funds on health commodities and 52% on health facility infrastructure (compared to a mean of 16% and 38% from other health centers). In 2018–2019, urban dispensaries reported the lowest expenditure on medical supplies and equipment, at only 3% of their total annual expenditure.

**Figure 2:**
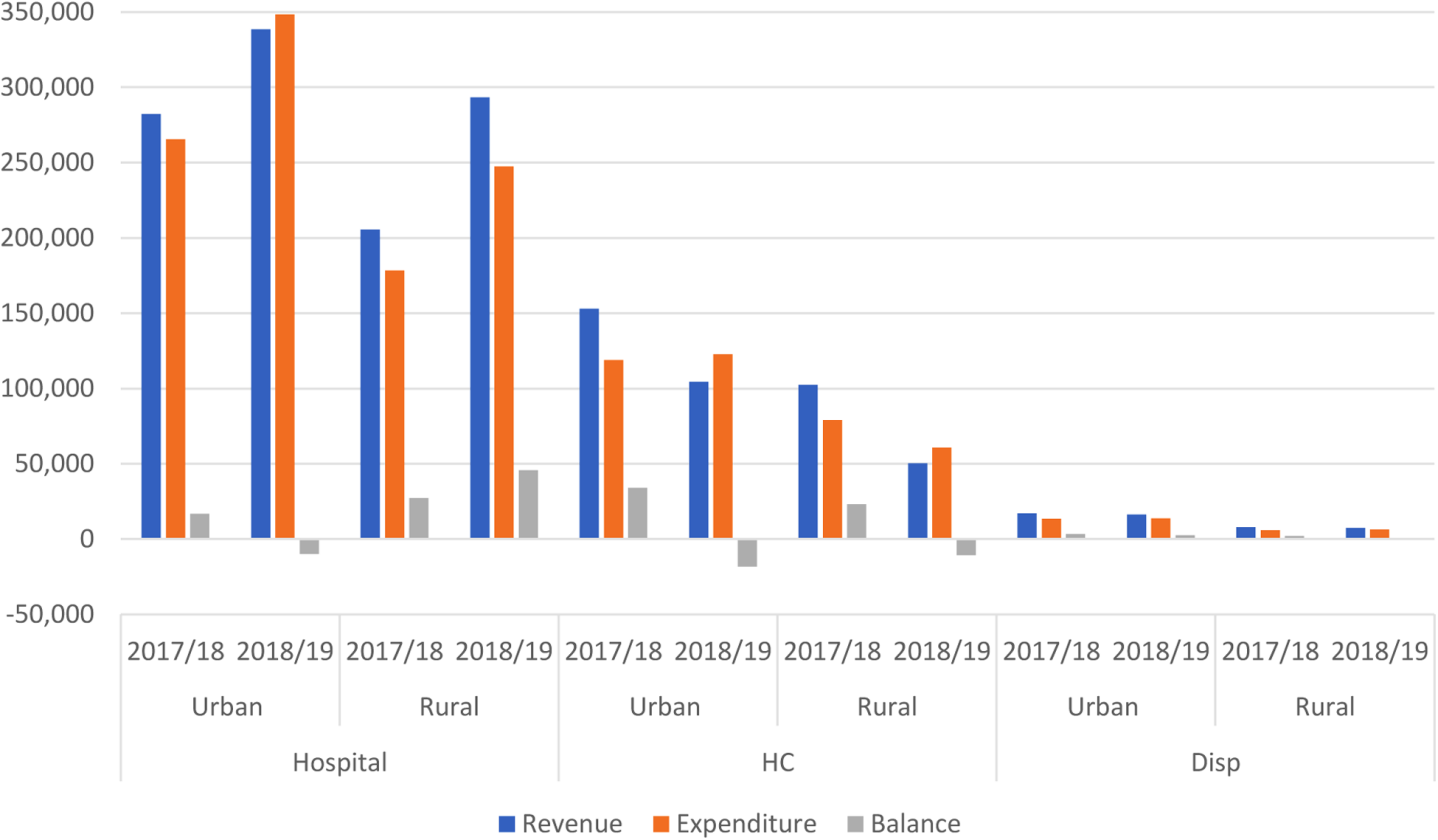
Revenue compared to expenditure by facility type and facility setting.

Comparing revenue to expenditure, most of the revenues were almost fully expended with a few exceptions noted in 2018–2019, when rural hospitals and health centers—both urban and rural—expended more than their revenue (Figure 2).

Key informant respondents ascertained to the fact that the FFARS has improved fund utilization at health facilities noting,

*Formerly, the fund processing took a long time as the District Executive Director (DED) could take long to authorize, but now funds go directly to facilities and to a large extent authorization is done at the facility…*

During these first years of the FFARS implementation, data quality may be questionable due to the lack of skill sets and shortage of supporting staff at lower-level health facilities, and system users do not necessarily use the appropriate procedures in data entry, generating errors and subsequently complicating reconciliations of the facilities’ accounts. A respondent from LGA had this to say:

*Sometimes facilities try to find a short cut… e.g., if they want to pay and find that the code does not have enough funds, they generate a new receipt instead of asking for assistance, which later brings a problem in reconciliation.*

Acceptability of the system dictates its use and ultimately quality data for decision making. As one of the respondents put it, most users now perceive them as tools to help them in their work and not as tools to control them or as a burden:

*When we started the perception was bad, it was perceived that it was a burden. Now they are generally taken well and it is appreciated that the systems are there to assist them and to make work easier*.

## Discussion

The aim of the present study was to assess the functionality of the FFARS in management, accounting, and reporting funds received and spent in the context of progress towards UHC. From the 2 years of FFARS implementation, we have observed that sources of revenue are predominantly from user fees and HSBFs, while expenditure was mainly drugs and medicines for hospitals and health facility infrastructure for health centers and dispensaries.

Even though the FFARS is implemented at the PHC level, the sources of revenue at this level portray the growing share of user fees as reported in the recent Tanzania Health Sector Public Expenditure Review 2020 (10). The predominance of user fees has been shown to hamper progress towards UHC (6). Health insurance coverage is also low. By December 2019, the National Health Insurance Fund covered 9% of the population (an increase from 2% approximately 20 years ago) while the improved Community Health Fund was reported to have reached a coverage of 23% of households (11, 12). However, a recent review has indicated that active membership, donating households with an active insurance policy, by April 2021 was only 3% (13).

Sustainable health financing for UHC requires more than just increased public spending—it also requires predictability and stability in these revenues. In turn, these attributes are enablers for greater efficiency in how the revenues are used (14, 15). With regard to predictability, the analysis revealed a decrease in revenue especially in health centers and dispensaries, which serve the majority of the poor communities at the grass root level. This could be a result of data entry error; however, declines of 30%–50% observed in health centers may require further analysis. In the FYs 2017–2018 and 2018–2019, Tanzania was yet to be declared a middle-income country and with that status donor support is expected to progressively decline (16). The over-reliance on HSBFs is another issue to look at as the country now needs to move towards more mobilization of domestic revenue (17).

During these first years of the FFARS implementation, its value may not necessarily be in tracking sources of revenue and expenditure but the mere ability to have access to such information in real time, which was a huge bottleneck before the introduction of the system. With the FFARS, financial reporting and data visibility have been made a reality, which has significantly improved the decision-making process. This value has been ascertained in another paper on the integration of health care and financial information: evaluation in a public hospital using a comprehensive approach that showed that the new integrated system improved the managers’ use of information, making it more accessible, reliable, and timely (18). Another study on the use of data in health policy and financing has indicated that quality data evidence is used for improved efficiency of financing and policymaking (19).

The success of these systems is closely tied to user-perceived usefulness (20). This study showed that respondents perceived the FFARS to be useful amid its initial challenges. Further studies after a few years of implementation are crucial to support the hypothesis of the user's perception of a greater fit between these systems and their needs (18).

As would have been expected with the implementation of a new system, several challenges were recorded. There is a notable shortage of ICT equipment, such as computers and printers, that complicates the utilization of the systems, compelling lower-level and rural facilities to utilize informal means such as Internet cafés and stationery shops to access the system and print FFARS vouchers and PlanRep reports. Inadequate electricity and Internet connectivity remain a bottleneck in the implementation and utilization of the systems. Responding to emerging needs through the provision of technical support is hampered by the help desk being stationed at the national level with limited capacity for this center to be able to effectively respond to all 185 LGAs and the more than 5,000 facilities with the current 80 requests for assistance each day. Some efforts to rectify this included a plan to recruit 100 ICT staff to be allocated at the national, regional, and council levels to provide capacity building to LGAs and establish help desks. Nevertheless, as the implementation of such electronic integrated information system advances, there is a progress in the reduction of time spent as human resource capacity improves and so the quality of the information improves (18).

## Conclusion

The implementation of the FFARS in Tanzania demonstrated its high potential in improving facility financial management, including its ability to track revenue and expenditure at PHC facilities. Staffing shortages, ICT infrastructure, and limited opportunities for capacity building could be the limiting factors to reaching the potential of the implementation of the FFARS and the attainment of its full impact on Tanzania's pursuit for UHC.

## Data Availability

The original contributions presented in the study are included in the article/Supplementary Material, further inquiries can be directed to the corresponding author.

## References

[1] Kutzin J Witter S Jowett M Bayarsaikhan D. Developing a national health financing strategy: A reference guide. Health financing guidance. Geneva: World Health Organization (2017).

[2] World Health Organization. Health systems financing: the path to universal health coverage: plan of action (2012). Geneva: World Health Organization. 10.2471/BLT.10.078741 PMC2878164 20539847

[3] Kapologwe NA Kalolo A Kibusi SM Chaula Z Nswilla A Teuscher T Understanding the implementation of direct health facility financing and its effect on health system performance in Tanzania: a non-controlled before and after mixed method study protocol. Health Res Policy Syst. (2019) 17(1):11. 10.1186/s12961-018-0400-3 30700308 PMC6354343

[4] World Health Organization. Sustainable health financing, universal coverage and social health insurance (2005). Geneva: World Health Organization.

[5] Leli H Addulahi O Tsofa B. Public hospitals' finance management systems, and accountability mechanisms in the context of decentralized health systems in low- and middle-income countries? A thematic review. Open Res Africa. (2019) 2(18):1–13. 10.12688/aasopenres.12962.1

[6] Ruhago GM Kapologwe NA Ngalesoni FN Kengia JT Kibusi SM Kalolo A Cost-efficiency analysis of the improved web-based planning, budgeting, and reporting system (PlanRep) in Tanzania. Front Health Serv. (2022) 1:1–15. 10.3389/frhs.2021.787894 PMC10012613 36926476

[7] Wang H Naghavi M Allen C Barber RM Bhutta ZA Carter A Global, regional, and national life expectancy, all-cause mortality, and cause-specific mortality for 249 causes of death, 1980–2015: a systematic analysis for the global burden of disease study 2015. Lancet. (2016) 388(10053):1459–544. 10.1016/S0140-6736(16)31012-1 27733281 PMC5388903

[8] Schoonenboom J Johnson RB. How to construct a mixed methods research design. Kolner Z Soz Sozpsychol. (2017) 69(Suppl 2):107–31. 10.1007/s11577-017-0454-1 28989188 PMC5602001

[9] Kapologwe NA Meara JG Kengia JT Sonda Y Gwajima D Alidina S Development and upgrading of public primary healthcare facilities with essential surgical services infrastructure: a strategy towards achieving universal health coverage in Tanzania. BMC Health Serv Res. (2020) 20(1):218. 10.1186/s12913-020-5057-2 32183797 PMC7076948

[10] World Bank. Tanzania health sector public expenditure review 2020 (2020). World Bank.

[11] Amu H Dickson KS Kumi-Kyereme A Darteh EKM Understanding variations in health insurance coverage in Ghana, Kenya, Nigeria, and Tanzania: evidence from demographic and health surveys. PLoS ONE. (2018) 13(8):e0201833. 10.1371/journal.pone.0201833 30080875 PMC6078306

[12] Amani PJ Hurtig AK Frumence G Kiwara AD Goicolea I San Sebastiån M Health insurance and health system (un) responsiveness: a qualitative study with elderly in rural Tanzania. BMC Health Serv Res. (2021) 21(1):1140. 10.1186/s12913-021-07144-2 34686182 PMC8532322

[13] Switzerland Development Cooperation. Mid-term review phase 3-exit phase-report of the health promotion and system strengthening (HPSS) project – Tanzania (2021). Switzerland Development Cooperation.

[14] Cashin C Bloom D Sparkes S Barroy H Kutzin J O'Dougherty S Aligning public financial management and health financing: Sustaining progress toward universal health coverage. Geneva: World Health Organization (2017).

[15] Junquera-Varela RF Verhoeven M Shukla GP Haven B Moreno-Dodson B. Strengthening domestic resource mobilization: Moving from theory to practice in low- and middle-income countries. Washington, DC: World Bank Publications (2017).

[16] Moyo M Simson R Jacob A De Mevius F. Attaining middle income status – Tanzania: growth and structural transformation required to reach middle income status by 2025. Proceedings of the Dar es Salaam, International Growth Centre, Tanzania Dar es salaam (2012). p. 31.

[17] Wright S. Effect of development assistance on domestic health expenditures. Lancet. (2010) 376(9741):590–1. 10.1016/S0140-6736(10)61291-3 20728748

[18] Escobar-Pérez B Escobar-Rodríguez T Bartual-Sopena L. Integration of healthcare and financial information: evaluation in a public hospital using a comprehensive approach. Health Informatics J. (2016) 22(4):878–96. 10.1177/1460458215595259 26276796

[19] Kumar MB Taegtmeyer M Madan J Ndima S Chikaphupha K Kea A How do decision-makers use evidence in community health policy and financing decisions? A qualitative study and conceptual framework in four African countries. Health Policy Plan. (2020) 35(7):799–809. 10.1093/heapol/czaa027 32516361 PMC7487332

[20] Kwahk K-Y Lee J-N. The role of readiness for change in ERP implementation: theoretical bases and empirical validation. Inf Manag. (2008) 45(7):474–81. 10.1016/j.im.2008.07.002

